# The profile of unusual beliefs associated with metacognitive thinking and attributional styles

**DOI:** 10.1002/pchj.528

**Published:** 2022-02-15

**Authors:** Elle P. Coleman, Rodney J. Croft, Emma Barkus

**Affiliations:** ^1^ School of Psychology University of Wollongong Wollongong New South Wales Australia; ^2^ Department of Psychology Northumbria University Newcastle upon Tyne UK; ^3^ Illawarra Health & Medical Research Institute Wollongong New South Wales Australia

**Keywords:** attributional styles, metacognitive thinking, psychosis continuum, unusual beliefs

## Abstract

Cognitive interpretations of daily events may differ in people from the general population who hold unusual beliefs. It is also important to understand whether different belief profiles exist to appreciate which patterns of beliefs are less psychologically healthy. Cluster analysis was used to form unusual belief profiles in a general population sample (*n* = 578; *M*_age_ = 22 years, *SD* = 6.98; 80% female) across paranoid, paranormal, and magical ideation beliefs, and we assessed whether they differed in attribution style and metacognitive beliefs about worry. Four clusters were formed: low on all measures (low all); high on all measures (high all); comparably higher on paranormal beliefs (paranormal group); and comparably higher on paranoid beliefs (paranoid group). For total Metacognitions Questionnaire‐30, the high all and high paranoid clusters did not differ, and both clusters scored higher than the high paranormal group, who all scored higher than the low all cluster. For attributional styles (Attributional Styles Questionnaire), lower scores on internal positive attribution were found for the high all and high paranoid clusters compared to the low all and high paranormal clusters. The high paranormal cluster had higher scores than the high paranoid cluster on self‐serving bias. Differences in attributional style appeared to be driven by mental health diagnosis. Our results suggest different profiles of unusual beliefs are detectable in the general population that differ in their metacognitive beliefs and perceived causation of events in their environment. Future studies investigating delusional proneness need to consider multiple unusual beliefs as well as assessing mood state and distress.

## INTRODUCTION

Cognitive theorists have proposed that delusions arise through biased evaluative and reasoning processes used in an attempt to make sense of everyday experiences (Garety & Freeman, 1999). Delusions are strongly held beliefs which lie outside of social norms, do not vary in conviction when challenged, and are heavily influenced by sociocultural experiences (Bell et al., 2006; Feyaerts et al., 2021). They are a core symptom of schizophrenia spectrum disorders and other psychopathology including major depressive disorder (Arciniegas, 2015; Keller et al., 2007). Importantly, delusional beliefs in patients with schizophrenia have been associated with poor social and emotional functioning and heightened levels of distress (Freeman & Garety, 1999). Given that the content of delusions often relates to one's place within the social world (Dutta et al., 2007; Kiran & Chaudhury, 2009), it follows that misinterpretation of social interactions and events will affect daily functioning (Garety et al., 2001; Morrison, 2001). Therefore, it is important to increase understanding of the cognitive processes that underpin the formation and maintenance of delusions.

Exaggerated cognitive biases (here referred to as “cognitive styles”), commonly seen in patients with delusions, are defined as systematic tendencies to perceive and interpret information differently, or adopt alternative styles of thinking when processing certain information (Bell et al., 2006; Moritz & Woodward, 2007). Metacognitive thinking and causal attribution biases are two major cognitive styles that have been suggested to underlie delusion formation and maintenance (Garety & Freeman, 2013).

Metacognitions represent a broad, sweeping set of constructs that reflect thinking about thinking (Wells & Carter, 2001). Cognitive processes, including worry, threat monitoring, and self‐regulatory processes, that do not appropriately modify unhelpful self‐knowledge are thought to be influenced by metacognitive beliefs (Goldstone et al., 2013; Wells & Matthews, 1996). Metacognition is commonly assessed using the Metacognitions Questionnaire–short form (MCQ‐30), which taps into metacognitive processes for worry including: cognitive self‐confidence, positive beliefs about worry, cognitive self‐consciousness, negative beliefs about uncontrollability of thoughts and danger, and beliefs about the need to control thoughts (Wells & Cartwright‐Hatton, 2004). The MCQ‐30 captures maladaptive self‐regulatory processes used to deal with increased thoughts about worry (see self‐regulatory executive functioning model; Wells, 1995). If metacognitive worry processes are used to self‐regulate, nonconfrontational situations are likely to be perceived as threatening, leading to greater distress and further use of worry to alleviate distress. Patients with schizophrenia who have delusions tend to report worry as uncontrollable and dangerous (e.g., “My worrying could make me go mad”), the need to control thoughts (e.g., “If I cannot control my thoughts it means I am going crazy”), and low cognitive confidence (e.g., “I have little confidence in my memory for words and names”), in comparison to healthy controls (Austin et al., 2015; Startup et al., 2016; Valiente et al., 2012). Therefore, people with delusions may engage in maladaptive self‐regulation strategies (i.e., metacognitive worry) that serve to maintain delusions and exacerbate distress.

Another cognitive process used to construct beliefs about the world is causal attribution. Causal attribution bias is characterized by retrospective explanations for event causation related to attributing positive events to internal factors (i.e., “An event happened because of me”) and negative events to external factors (i.e., “An event happened because of something outside of me”), or a combination of both, known as self‐serving bias (SSB) (Bentall et al., 1991; Bentall et al., 1994; Heider, 2013; Kinderman & Bentall, 1997). In patients with schizophrenia, SSB is exaggerated in comparison to healthy controls, and is particularly prevalent in those persons with paranoid delusions (Müller et al., 2021), possibly protecting against low self‐esteem (Bentall et al., 1994). Thus, consistently held SSB may lead to distorted perceptions of the social world as a hostile environment, fostering delusional ideation.

It is clear that cognitive styles have some role to play in the formation and maintenance of delusions. Indeed, cognitive therapies that target these biases decrease the severity of delusions in patient samples (Gawęda, Krężołek, et al., 2015; Kumar et al., 2010; Mehl et al., 2015). Even so, examining cognitive styles in patients is complicated by the presence of comorbid symptoms associated with clinical disorder diagnoses. This makes it difficult to tease apart the extent to which cognitive styles impact on delusion formation and maintenance outside of other symptoms. In addition, to substantiate the predictive validity of cognitive styles for delusional beliefs, cognitive styles would need to be present prior to a diagnosis of clinical delusion. To account for these issues, there is promise in assessing cognitive styles in nonpatients who hold beliefs that resemble delusions.

A psychosis continuum hypothesis is based on the assumption that delusion‐like characteristics present in the general population and reflect a vulnerability to clinical delusions (McGrath et al., 2015; Van Os et al., 2009). Unusual beliefs are those beliefs that resemble delusions in nature, but are held by people without a diagnosis of a clinical disorder (Scott et al., 2006). Like delusions, unusual beliefs sit outside social norms, are held with strong conviction despite existence of contrary evidence, and are often associated with feelings of distress (Varghese et al., 2011). A core assumption of the psychosis continuum hypothesis is that people who hold unusual beliefs are also likely to hold cognitive biases similar to persons with delusions, placing them at a higher risk of experiencing clinical delusions (Van Os et al., 2009). If unusual beliefs are held inflexibly, regardless of their content, they have the potential to be unhelpful. However, there remains a lack of consensus around the existence and nature of a psychosis continuum in relation to unusual beliefs (Lawrie et al., 2010; Linscott & Van Os, 2013). If the continuum theory holds, cognitive styles seen in patients with delusions should also be present in a similar, albeit attenuated form, in psychologically healthy people who endorse unusual beliefs.

Self‐report questionnaires are used to capture unusual belief themes, including paranoid thinking (Green et al., 2008), paranormal beliefs (Tobacyk & Milford, 1983), and magical ideation (Eckblad & Chapman, 1983). A broad unusual beliefs construct is considered multidimensional to comprise separable unusual beliefs such as suspiciousness, paranoia, magical ideation, and paranormal beliefs. For example, Green et al.’s (2008) Paranoid Thoughts Scale measures paranoia for social reference (personally internalized communications or observations that may not be objectively related to the self) and persecutory thinking (the belief that harm is occurring or will occur to them and that the persecutor intends to cause harm). In contrast, the 26‐item Revised Paranormal Belief Scale (Tobacyk, 2004) captures belief in the paranormal that, if genuine, would violate basic limiting principles of science (e.g., believing that a mental event can directly affect a physical event). Finally, the Magical Ideation Scale (MIS) assesses magical ideation, defined as those “beliefs and reported experiences in forms of causation that by conventional standards are invalid” (Eckblad & Chapman, 1983, p. 215). Although magical ideation is considered a measure of schizotypy as defined by Meehl (1964), it captures only one component of several which comprise schizotypy more broadly. Indeed, Hergovich et al. (2008) were not able to subsume the MIS under the Schizotypal Personality Questionnaire (SPQ; Raine, 1991) nor the 26‐item revised Paranormal Beliefs Scale (Tobacyk, 2004) in an adolescent sample. Magical ideation, unlike paranormal beliefs, tends to be self‐referential, used to provide context and meaning for self experiences, whereas paranormal beliefs refer to the existence of possible experiences and abilities without the responder necesarily having personal experience of the phenomena. Despite the high correlation between scales for magical ideation and paranormal beliefs, they are not interchangeable (Day & Peters, 1999; Thalbourne, 1984, 1994; Thalbourne & French, 1995). Accumulating evidence has suggested that despite any content similarity, magical ideation, paranormal beliefs, and paranoid thoughts are justifiably considered separate constructs and worthy of consideration within one study.

The relationship between metacognitive styles and unusual beliefs has been investigated in community samples. Negative beliefs surrounding threat or lack of control have been associated with persecutory and suspicious ideas, paranormal beliefs, and delusion proneness in general, even when accounting for hallucination proneness (Brett et al., 2009; Bright et al., 2018; Goldstone et al., 2013; Larøi & Van Der Linden, 2005). Cognitive confidence and uncontrollability were reported to be associated with paranormal beliefs, but this was for females only (Irwin, 2012) whereas some studies have reported no association between metacognitive biases and unusual beliefs in nonclinical samples (Brett et al., 2009; Bright et al., 2018; Goldstone et al., 2013; Larøi & Van Der Linden, 2005). Mixed findings concerning metacognitive thinking in people who hold unusual beliefs have suggested that it is worthy of additional consideration.

Different types of attributional bias have also been investigated in psychologically healthy people who hold unusual beliefs. Externalization of negative events, internalization of positive events, and SSB have been associated with unusual beliefs in some studies (Gawęda, Prochwicz, et al., 2015; So et al., 2015), but not in others (Janssen et al., 2006; Martin & Penn, 2001; McKay et al., 2005). The variation of findings could be related to measurement differences (Mehl et al., 2014), or it may be that attributional biases only manifest when delusions are of a clinical nature (Martin & Penn, 2001; McKay et al., 2005). Failing to find consistent relationships between cognitive styles and healthy people holding unusual beliefs questions whether a continuum model of delusions exists. However, past studies focusing on a single belief have not accounted for the likelihood that people can hold multiple unusual beliefs at a time (Kiran & Chaudhury, 2009). This precludes the possibility that particular belief profiles (i.e., the expression of multiple unusual beliefs) could be associated with different cognitive styles.

Cluster analysis provides an opportunity to consider an individual's pattern of beliefs across multiple measures to reveal participant groups or clusters characterized by belief profiles (Barrantes‐Vidal et al., 2003). This is in contrast to data‐reduction techniques that address interrelationships between items or measures and thus are ill‐equipped to explain how participants group. K‐means clustering partitions (n) cases into prespecified clusters (k) through maximizing between‐cluster difference and minimizing within‐cluster variance on prespecified variables (Hartigan, 1975). This includes an iterative process that allows cases to be reclassified into another cluster after the initial iteration if it provides a better fit (Kaufman & Rousseeuw, 2009). So far, cluster analysis has been used to characterize samples on psychosis proneness more broadly (Barrantes‐Vidal et al., 2003; Suhr & Spitznagel, 2001a, 2001b). These papers have consistently yielded four‐cluster models characterized by higher scores across all measures, lower scores across all measures (consistent with expectation from a general population sample), and positive and negative schizotypy. However, no study to date has used a cluster analysis technique to assess an individual's patterns of responses across multiple unusual belief measures.

In summary, the psychosis continuum model holds that psychologically healthy people with unusual beliefs should have similar patterns of cognitive styles held by patients with delusions. One way of evaluating the adequacy of the continuum model is to assess whether there are relationships between cognitive styles and unusual beliefs in healthy people. Therefore, the aim of this study was to assess cognitive styles of thinking and holding multiple unusual beliefs concurrently in a male and female general population sample. Grouping together different unusual beliefs, rather than investigating only a single unusual belief, may more closely reflect what occurs for people in the real world, increasing the ecological validity of findings. In doing so, this study will demonstrate how endorsing more than one set of unusual beliefs characterizes cognitive styles implicated in the formation of unusual beliefs. We hypothesize that four meaningful groupings of unusual belief profiles will emerge using k‐means clustering. We also hypothesize that participants with higher endorsement of unusual beliefs will show greater maladaptive cognitive styles. Specifically, clusters with higher endorsement of unusual beliefs as compared to those with lower levels of beliefs will have higher maladaptive metacognitive styles. Finally, persons who endorse unusual beliefs will have external attributional biases for negative events, internalization of positive events, and an exaggerated SSB in comparison to those with lower endorsement of unusual beliefs.

## METHODS

### Participants

Participants were 578 (*M*_age_: 22 years, *SD*: 6.98; 80% female) undergraduate students and general community people from in and around the University of Wollongong, Australia (UoW), recruited though research participation and via word of mouth. There were 153 cases (26.5%) of persons currently diagnosed with a mental health disorder that primarily comprised of anxiety disorders (33%) and comorbid anxiety and depression (30%). The remaining 40% consisted of disorders of behavior, eating, development, personality, mood, posttraumatic stress, and depression. All UoW participants received university course credit for participation. There were 195 cases of reported mental health help‐seeking within the 6 months prior to study participation. No reimbursement was offered for non‐UoW participants.

### Materials

#### Demographics

A demographics questionnaire was created to best capture potentially confounding variables, including age, gender, history of mental health diagnosis, and mental health help‐seeking within the past 6 months.

#### Cognitive style measures

##### Metacognition

Metacognition was measured using the MCQ‐30 (Wells & Cartwright‐Hatton, 2004). This scale assesses maladaptive metacognitive beliefs related to worry processes and cognitive monitoring strategies. Higher scores on this questionnaire indicate a vulnerability to heightened distress associated with thoughts. The MCQ‐30 has a five‐factor structure which includes the subscales Cognitive Self‐Confidence (CSC), Positive Beliefs About Worry (POS), Cognitive Self‐Consciousness (CC), Negative Beliefs About Uncontrollability of Thoughts and Danger (NEG), and Beliefs About Need To Control Thoughts (NC). Participants respond on a 4‐point Likert scale from 1 (*do not agree*) to 4 (*agree very much*), indicating the degree to which the item applied to themselves. The MCQ‐30 has shown good internal consistency, convergent validity, test–retest reliability, and cross‐cultural reliability (Ramos‐Cejudo et al., 2013; Wells & Cartwright‐Hatton, 2004; Zhang et al., 2020). Internal consistency in the current sample was excellent, with Chronbach's α = .917.

##### Causal attribution

The Attributional Styles Questionnaire (ASQ; Peterson et al., 1982) assesses responses to 12 hypothetical situations (six positive, six negative) that tap into individual differences in the use of the following attributional dimensions: internal versus external, stable versus unstable, and global versus specific causes of events. Participants are asked to write down one major cause they would attribute to the occurrence of the specified event. They are then required to answer on a 7‐point Likert scale whether the situation occurred from 1 (*totally due to other people or circumstances*) to 7 (*totally due to me*), whether the cause 1 (*will never be present again*) or 7 (*will always be present*), and whether the cause is something that 1 (*influences just this particular situation*) or 7 (*influences all situations*). Higher scores indicate internalization of events whereas lower scores indicate externalization of events. Items pertaining to globality and stability of events have been collected, but are not reported here due to limited relevance to psychosis proneness research (Jolley et al., 2006). SSB was calculated as the negative mean minus the positive mean for internal versus external causes of events. A larger difference indicates greater SSB. There have been reported issues with internal consistency (Kinderman & Bentall, 1997); however, Chronbach's α within the current sample was adequate at .763.

### Unusual beliefs measures

#### Magical ideation

The 30‐item Magical Ideation Scale (MIS; Eckblad & Chapman, 1983) captures magical thinking, defined as belief in unconventional causal explanations for events. Binary responses of 1 (*true*) or 0 (*false*) are used to indicate endorsement of each item. The MIS has good internal consistency, test–retest reliability, and cross‐cultural validity (Atbaşoğlu et al., 2003; Barnes & Nelson, 1994; Fonseca Pedrero et al., 2009). A Chronbach's α of .848 shows good internal consistency in this sample.

#### Paranormal beliefs

The 26‐item Revised Paranormal Beliefs Scale (RPBS; Tobacyk, 2004) was used to assess belief in religiosity and the paranormal. Subscale dimensions of traditional religious beliefs, psi, witchcraft, superstition, spiritualism, extraordinary life forms, and precognition are responded to on a 7‐point Likert scale from 1 (*strongly disagree*) to 7 (*strongly agree*), where higher scores indicate endorsement of the subscale. The RPBS shows good psychometric integrity (Drinkwater et al., 2017) and excellent internal consistency in the current sample, Chronbach's α = .921.

#### Paranoid thoughts

Green et al.’s (2008) Paranoid Thoughts Scale (GPTS) provides a valid and reliable assessment of paranoid thought divided into two 16‐item subscales representing ideas of social reference and persecution. Items of each subscale are responded to using a 5‐point Likert format from 1 (*not at all*) to 5 (*totally agree*), where higher scores reflect endorsement of each item within the subscale. A Chronbach's α of .960 indicates excellent internal consistency in this sample.

#### Procedure

Participants completed all questionnaires online using the survey platform Survey Monkey (http://surveymonkey.com). Data were collected over a 5‐month period from October 2018 to March 2019. This study was approved by the UoW Social Science and Humanities Human Research Ethics Committee (#2018/431), and informed consent was provided by all participants.

#### Data analysis

We ran a k‐means cluster analysis using total scores on the MIS, RPBS, and GPTS as participant grouping variables. The appropriate number of clusters was specified according to where a balance was drawn between minimal within‐cluster variance and maximized between‐cluster difference after limited iterations (<15; Kaufman & Rousseeuw, 2009). A one‐way multivariate analysis of variance (MANOVA) with the clusters as the independent variable and unusual beliefs scores as the dependent variables was used to assess the appropriateness of the cluster solution. One‐way ANOVAs were used to ensure that clusters differed on the basis of their presentation of unusual beliefs. We compared cluster profiles on demographic variables, including age, sex, status of current mental health diagnosis, and whether mental health help had been sought in the past 6 months, using the Pearson χ^2^ test for categorical variables and ANOVAs for continuous dependent variables where appropriate. A MANOVA was performed to test for an effect of MCQ‐30 on cluster profiles, taking into account the effect of mental health diagnosis, via a 5 × 4 × 2 (MCQ‐30 × Cluster × Mental Health Diagnosis) design. A second MANOVA was performed to test for an effect of ASQ on cluster profiles, taking into account the effect of mental health diagnosis, via a 3 × 4 × 2 (ASQ × Cluster × Mental Health Diagnosis) design. Bonferroni corrections were applied to all post hoc analyses.

## RESULTS

### Cluster analysis and cluster profiles

A k‐means cluster analysis was performed using three‐, four‐, five‐, and six‐cluster solutions, to identify data‐driven groupings of participants based on their scores on the MIS, RPBS, and GPTS. Here, the best solution was provided by the use of four clusters because it yielded optimal balance between within‐cluster homogeneity and between‐cluster heterogeneity after eight iterations, showed less iterations‐to‐convergence than three‐, five‐, and six‐cluster solutions, and limited nonsignificant difference between clusters across each unusual belief dimension on belief presentation after controlling for Bonferroni multiple comparisons (Table 1). Three clusters also showed an adequate solution, particularly as all clusters differed significantly across each unusual belief dimension on belief presentation. However, the three‐cluster solution required more iterations to convergence and had lower effect sizes on all unusual belief measures in comparison to a four‐cluster solution. Therefore, in line with previous literature (Barrantes‐Vidal et al., 2003; Suhr & Spitznagel, 2001a, 2001b), a four‐cluster solution was chosen to demonstrate how groupings of unusual beliefs are related to cognitive styles implicated in the formation and maintenance of unusual beliefs (for *f* values, see Table 1).

**TABLE 1:** Difference between clusters across each unusual belief dimension on belief presentation

Clusters	Unusual belief dimensions	*F* (*df*)	*p*	η_p_^2^	Cluster difference
Three clusters	MIS	136.954 (2, 575)	<.001	.323	
11 Iterations	RPBS	496.838 (2, 575)	<.001	.633	
	GPTS	471.683 (2, 575)	<.001	.621	
Four clusters	MIS	133.428 (3, 574)	<.001	.461	No diff: 2, 4
8 Iterations	RPBS	564.923 (3, 574)	<.001	.772	
	GPTS	424.513 (3, 574)	<.001	.719	
Five clusters	MIS	107.187 (4, 573)	<.001	.428	No diff: 1, 5; 4, 5
11 Iterations	RPBS	533.124 (4, 573)	<.001	.788	No diff: 1, 2; 4, 3
	GPTS	350.005 (4, 573)	<.001	.710	No diff: 1, 3
Six clusters	MIS	89.463 (5, 572)	<.001	.439	
12 Iterations	RPBS	701.174 (5, 572)	<.001	.860	
	GPTS	310.014 (5, 572)	<.001	.730	No diff: 1, 2; 4, 5; 5, 6

*Note*: Cluster difference: identifying the clusters that do not differ across unusual belief dimension. For the four‐cluster solution, group means across unusual belief measures significantly differed from one another at *p* = < .05, except the high all and high GPTS cluster on MIS scores, *p* = 1.000, after controlling for multiple comparisons.

Abbreviations: GPTS = Green et al. Paranoid Thoughts Scale; MIS, Magical Ideation Scale; RPBS, Revised Paranormal Beliefs Scale.

**FIGURE 1:**
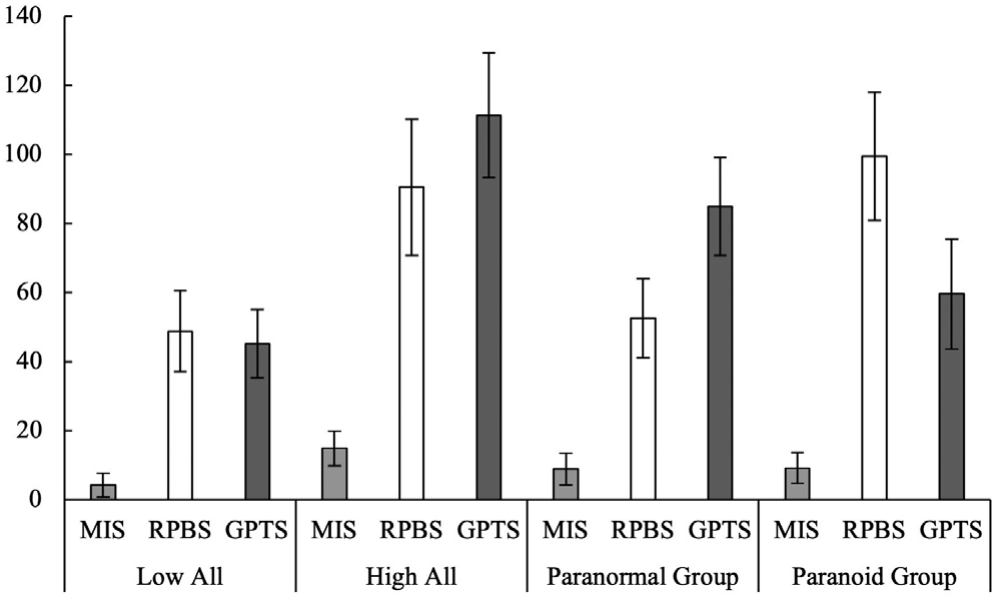
Mean cluster differences based on participant presentation of unusual beliefs (*n* = 578). Highest possible score for each unusual belief measure if all items are endorsed is as follows: Magical Ideation Scale (MIS) = 60, Revised Paranormal Beliefs Scale (RPBS) = 180, Green et al. Paranoid Thoughts Scale (GPTS) = 160. Error bars represent *SD* of the mean for between‐group profiles across unusual belief measures. *p* < .05

A discriminative index for clusters was created by running a MANOVA, with the clusters as the independent variable and unusual beliefs scores as the dependent variable (Barrantes‐Vidal et al., 2003). A significant Wilks's Λ demonstrated that only 20% of the total variability was left unexplained, Λ = .199, *p* = <.001, indicating that a four‐factor cluster solution was appropriate for the sample. Each group's profile of means and *SD*s are presented in Figure 1.

Cluster 1 contained 267 subjects who had low scores on paranoid, paranormal beliefs, and magical ideation, subsequently labeled the “low all” group. Cluster 2 consisted of 60 people who had higher scores on measures of paranoid beliefs, with a slightly higher predominance of paranormal beliefs and magical ideation scores compared to all other clusters; therefore, Cluster 2 was labeled the “high all” group. Cluster 3 represented 147 persons who had a higher than average score on paranormal beliefs, a moderate score on paranoid beliefs that was higher than the “low all” cluster, but lower than Cluster 4, and a below‐average score on magical ideation and so was named “paranormal group.” Finally, Cluster 4 comprised 85 people with higher paranoia belief scores than all other clusters, paranormal belief scores lower than Cluster 3, but higher than Cluster 1, and below‐average magical ideation scores and was so called the “paranoid group.” For the remainder of the article, each cluster will be interchangeably referred to as both a “group” and “cluster.”

### Demographic differences between clusters

Demographic characteristics of the four clusters and statistical results for overall group differences are displayed in Table 2 whereas details of subgroup analyses are described next.

**TABLE 2:** Demographic differences between clusters including descriptive statistics, between‐group differences, and post hoc tests across unusual belief clusters

	Cluster 1 Low all	Cluster 2 High all	Cluster 3 Paranormal group	Cluster 4 Paranoid group	Statistic value	*p*	Cluster difference^a^, *p*
	*n* = 267	*n* = 60	*n* = 147	*n* = 104			
	*M* (*SD*)	*M* (*SD*)	*M* (*SD*)	*M* (*SD*)	*df*		
Gender^b^ (% Female)	75.3%	85.0%	86.4%	81.7%	*χ* ^2^ 20.793 (9)	.014	ns
MHD^c^	20.6%	33.3%	24.5%	40.4%	*χ* ^2^ 39.537 (3)	<.001	1 < 2, 4; 4 > 1, 3
Anxiety	8.2%	11.6%	8.1%	7.7%			
Depression	1.5%	5%	2%	4.8%			
CAD	5.9%	11.6%	9.5%	11.5%			
BD	0.4%	1.6%	0%	4.8%			
ED	0.4%	0%	0%	0.9%			
Dev	0.4%	0%	0%	0%			
Mood	1.5%	0%	2%	3.8%			
PTSD	1.1%	3.3%	2%	3.8%			
PD	0.4%	3.3%	1.3%	2.8%			
6 Month MHH^d^	29.6%	43.3%	31.3%	42.3%	*χ* ^2^ 19.016 (3)	<.001	1 < 2, 4
Age	22.44 (8.179)	20.68 (4.386)	21.23 (6.792)	20.50 (4.457)	*F* 2.750 (3)	.042	ns

Abbreviations: BD, behavioral disorder; CAD, comorbid anxiety and depression; Dev, developmental disorder; ED, eating disorder; Mood, mood disorder; ns, nonsignificant; PTSD, posttraumatic stress disorder; PD, personality disorder.

^a^ Chi‐square post hoc tests show which clusters differed significantly at *p* = .05.

^b^ Cluster 3 includes *n* = 1 “Other Identifying” and *n* = 2 “Transgender” persons.

^c^ Percentage of within‐cluster number of cases of persons with a current mental health diagnosis (MHD).

^d^ Percentage of within‐cluster number of cases of persons who have sought help for mental health purposes in the past 6 months (6 Month MHH).

People with a current mental health diagnosis were more likely to be in the paranoid cluster compared to the low all cluster, χ^2^[1, N = 104] = 15.173, *p* = .001, and the paranormal cluster, χ^2^[1, N = 147] = 7.184, *p* = .007. Reports of mental health help seeking within the past 6 months were equally as likely to have come from the high All cluster as the paranoid group, and both of those clusters were more likely to seek mental health help than those in the low all cluster, high all: χ^2^[1, N = 62] = 4.246, *p* = .039; paranoid group: χ^2^[1, N = 104] = 5.464, *p* = .019. Although there was an overall main effect for age, post hoc comparisons between the clusters were not significant. The low all cluster had the highest average age whereas the paranoid group had the youngest age; however, this was only a difference of 1.94 years. There were no significant differences in the distribution of gender across the clusters after controlling for multiple comparisons.

### Main effect and interaction of cognitive measures and mental health diagnosis on cluster profiles

Tables 3 and 4 represent estimated marginal means at *p* = .05 significance for main effect and interactions, respectively. Because the difference in age between the clusters was marginal, this was not included as a covariate in the analysis. Because previous research has suggested that mental health status is significant for cognitive biases, and there were differences in the distribution of those with a mental health disorder status, this was placed as an independent variable in the subsequent analysis. Help‐seeking for mental health symptoms in the past 6 months was also distirubuted differently across our clusters; however, this is a more ambiguous question and was therefore used as a covariate in subsequent analysis.

**TABLE 3:** Descriptive statistics, estimated marginal means, and *SE*s for cluster and mental health diagnosis across cognitive styles measures

	MCQ‐30					ASQ		
	POS	NEG	CC	NC	CSC	NEG	POS	SSB
	*M* (*SE*)	*M* (*SE*)	*M* (*SE*)	*M* (*SE*)	*M* (*SE*)	*M* (*SE*)	*M* (*SE*)	*M* (*SE*)
Cluster								
Low all (*n* = 265)	13.760 (.261)	12.562 (.244)	13.129 (.276)	11.190 (.238)	12.100 (.273)	4.595 (.074)	5.097 (.071)	−.502 (.107)
High all (*n* = 62)	16.911 (.457)	15.355 (.428)	16.049 (.483)	13.765 (.416)	15.309 (.477)	4.474 (.121)	4.667 (.124)	−.193 (.187)
Paranormal cluster (*n* = 147)	14.260 (.325)	13.475 (.304)	13.547 (.344)	11.738 (.296)	12.494 (.340)	4.441 (.092)	5.045 (.088)	−.604 (.133)
Paranoid cluster (*n* = 104)	16.879 (.339)	15.531 (.317)	16.001 (.358)	14.080 (.308)	15.228 (.354)	4.725 (.096)	4.772 (.092)	−.047 (.139)
								
MHD	16.118 (.331)	14.818 (.310)	15.231 (.350)	13.030 (.301)	14.456 (.346)	4.751 (.094)	4.753 (.090)	−.670 (.083)
No MHD	14.787 (.202)	13.644 (.189)	14.132 (.214)	12.356 (.184)	13.100 (.211)	4.367 (.057)	5.038 (.055)	−.002 (.136)

*Note*: Reported means are estimated marginal means and their *SE*s.

Abbreviations*: ASQ Subscales*: NEG, Internalization of Negative Events; POS, Internalization of Positive Events; SSB, Self‐Serving Bias. *MCQ‐30 Subscales*: CC, Cognitive Self‐Consciousness; CSC, Cognitive Self‐Confidence; NC, Beliefs About Need To Control Thoughts; NEG, Negative Beliefs About Uncontrollability of Thoughts and Danger; POS, Positive Beliefs About Worry. MHD, mental health diagnosis.

**TABLE 4:** Descriptive statistics, estimated marginal means, and *SE*s on measures of cognitive styles between reported mental health diagnosis

Measure subscale	Low all		High all		Paranormal group		Paranoid group		
	(*n* = 265)		(*n* = 62)		(*n* = 147)		(*n* = 104)		
	*M* (*SE*)		*M* (*SE*)		*M* (*SE*)		*M* (*SE*)		
	MHD	No MHD	MHD	No MHD	MHD	No MHD	MHD	No MHD	
	*n* = 211	*n* = 54	*n* = 41	*n* = 21	*n* = 111	*n* = 36	*n* = 62	*n* = 42	
MCQ‐30									MCQ‐30
POS	12.550 (.231)	14.981 (.457)	16.220 (.524)	17.619 (.732)	13.919 (.318)	14.611 (.559)	16.435 (.426)	17.333 (.518)	14.452 (.183)
NEG	11.697 (.216)	13.444 (.427)	14.927 (.490)	15.810 (.685)	12.883 (.298)	14.083 (.523)	15.032 (.399)	16.048 (.484)	14.231 (.171)
CC	12.199 (.244)	14.037 (.482)	15.683 (.554)	16.381 (.774)	13.531 (.336)	13.772 (.591)	15.449 (.450)	16.643 (.547)	14.681 (.193)
NC	10.592 (.210)	11.704 (.416)	13.976 (.477)	13.429 (.666)	11.541 (.290)	11.861 (.509)	13.484 (.388)	14.595 (.471)	12.693 (.166)
CSC	10.863 (.241)	13.426 (.477)	14.512 (.547)	16.238 (.765)	12.234 (.333)	12.833 (.584)	14.613 (.445)	14.929 (.541)	13.783 (.191)
ASQ									ASQ
NEG	4.412 (.065)	4.799 (.129)	4.187 (.148)	4.794 (.207)	4.347 (.090)	4.556 (.158)	4.478 (.121)	4.992 (.121)	4.559 (.052)
POS	5.169 (.063)	5.031 (.124)	4.850 (.142)	4.492 (.199)	5.123 (.087)	4.972 (.152)	4.997 (.116)	4.552 (.141)	5.895 (.050)
SSB	−.757 (.095)	−.231 (.187)	−.663 (.215)	.302 (.300)	−.776 (.131)	−.417 (.229)	−.519 (.175)	−.440 (.212)	−.336 (.075)

*Note*: Reported means are estimated marginal means and their *SE*s.

Abbreviations: *ASQ Subscales*: NEG, Internalization of Negative Events; POS, Internalization of Positive Events; SSB, Self‐Serving Bias. *MCQ‐30 Subscales*: CC, Cognitive Self‐Consciousness; CSC, Cognitive Self‐Confidence; NC, Beliefs About Need To Control Thoughts; NEG, Negative Beliefs About Uncontrollability of Thoughts and Danger; POS, Positive Beliefs About Worry. MHD, mental health diagnosis.

### Main effects of cluster profile and mental health diagnosis on metacognitive beliefs

There was a significant main effect of cluster profile on all MCQ‐30 subscales, POS: *F* (3, 569) = 26.088, *p*  < .001, η_p_^2^ = .121; NEG: *F* (3, 569) = 23.952, *p* < .001, η_p_^2^ = .121; CC: *F* (3, 569) = 20.020, *p* < .001, η_p_^2^ = .095; NC: *F* (3, 569) = 24.386, *p* < .001, η_p_^2^ = .114; CSC: *F*  (3, 569) = 24.810, *p* < .001, η_p_^2^ = .116. Post hoc analyses showed the high all and paranoid group had higher scores than the low all and paranormal group across all MCQ‐30 subscales, *p* < .001. However, the high all and paranoid group did not differ from one another.

There was a significant main effect of mental health diagnosis on the POS, *F* (3, 569) = 10.569, *p* < .001, η_p_^2^ = .018; NEG, *F* (3, 569) = 9.394, *p* < .001, η_p_^2^ = .018; and CSC, *F* (3, 569) = 10.203, *p* < .001, η_p_^2^ = .018, MCQ‐30 subscales. Post hoc analyses showed current mental health diagnosis was associated with higher scores across the POS, NEG, and CSC MCQ‐30 subscales as compared to those with no mental health diagnosis.

The inclusion of mental health help‐seeking within the past 6 months as a covariate in the MANOVA model was nonsignificant.

### Cluster × Mental Health Diagnosis for metacognitive beliefs

The interaction between cluster and mental health diagnosis was nonsignificant across all MCQ‐30 clusters.

### Main effect of cluster profile and mental health diagnosis on causal attributional style

We observed a main effect of cluster profile on positive internalization, *F* (3, 569) = 4.929, *p* = .002, η_p_^2^ = .025, and SSB, *F* (3, 569) = 3.702, *p* = .012, η_p_^2^ = .019. The main effect of cluster profile on internalization of negative events was nonsignificant. Post hoc analyses showed that the low all group was more likely to internalize the cause of positive events as compared to the high all group, *p* = .014, and paranoid group, *p* = .028. The paranoid group had a diminished SSB as compared to the paranormal group, *p* = .022.

The main effect of all mental health diagnosis on ASQ subscales was significant, negative internalization: *F* (3, 569) = 19.354 , *p* = < .001, η_p_^2^ = .019; positive internalization: *F* (3, 569) = 6.541, *p* = .011, η_p_^2^ = .011; SSB: *F*(3, 569) = 15.847, *p* = < .001, η_p_^2^ = .027. Post hoc analyses demonstrated that greater internalization of negative events, *p* < .001, greater externalization of positive events, *p* = .011, and a diminished SSB, *p* < .001, was present in persons with a mental health diagnosis compared to those with no diagnosis.

Inclusion of mental health help‐seeking over the past 6 months as a covariate in the MANOVA model was nonsignificant.

### Cluster × Mental Health Diagnosis for attributional style

The interaction between mental health diagnosis and cluster on ASQ subscales was nonsignificant.

### Exploratory analyses

#### Effect of current mental health diagnosis between clusters across cognitive styles

Due to the significant main effect of mental health diagnosis, two subsequent MANOVAs (IV: Cluster; DV: Cognitive style subscales) split‐file by mental health diagnosis were conducted to assess the effect of self‐reported diagnosis and nondiagnosis within the sample. All reported means are estimated marginal means and their *SE*s.

#### Effect of mental health diagnosis between clusters on metacognitive beliefs

There was a significant main effect of cluster across all MCQ‐30 subscales in the nondiagnosed group: POS: *M* = 14.778, *SE* = .193, *F* (3, 421) = 30.474, *p* < .001, η_p_^2^ = .178; NEG: *M* = 13.631, *SE* = .180, *F* (3, 421) = 26.444, *p* < .001, η_p_^2^ = .159; CC: *M* = 14.142, *SE* = .207, *F* (3, 421) = 19.798, *p* < .001, η_p_^2^ = .124; NC: *M* = 12.402, *SE* = .169, *F* (3, 421) = 26.453, *p <* .001, η_p_^2^ = .159; CSC: *M* = 13.048, *SE* = .197, *F* (3, 421) = 27.863, *p* < .001, η_p_^2^ = .166, and the diagnosed group, POS: *M* = 16.138, *SE* = .299, *F* (3, 149) = 6.986 , *p* < .001, η_p_^2^ = .123; NEG: *M* = 14.848, *SE* = .284, *F* (3, 149) = 6.120 , *p* < .001, η_p_^2^ = .110; CC: *M* = 15.199, *SE* = .305, *F* (3, 149) = 6.959, *p* < .001, η_p_^2^ = .123; NC: *M* = 12.901, *SE* = .297, *F* (3, 149) = 6.691 , *p* < .001, η_p_^2^ = .119; CSC: M = 14.606, SE = .330, *F* (3, 149) = 7.005, *p* < .001, η_p_^2^ = .124.

Bonferroni‐corrected post hoc comparisons showed that for those without a mental health diagnosis, the high all group and paranoid group had higher scores across all mcq‐30 subscales compared with the low all and paranormal group, NEG: paranormal group < high all, *p* = .002; CC: paranormal group < high all: *p* = .002, paranormal group < paranoid group: *p* = .003; CSC: paranormal group < high all: *p* = .002. The paranormal group scored higher than the low all group across all MCQ‐30 subscales, POS: *p* = .003; NEG: *p* = .007; CC: *p* = .035; NC: *p* = .033; CSC: *p* = .004. All groups differed at *p* < .001 unless otherwise stated.

For those with a reported mental health diagnosis, the high all and paranoid group scored higher than the low all, high all: *p* = .022; paranoid group: *p* = .008, and paranormal group, high all: *p* = < .001; paranoid group: *p* = .004, on the POS subscale. The high all, *p* = .036, and paranoid group, *p* = .001, had higher NEG scores than the low all group. The high all, *p* = .042, and paranoid group, *p* = .003, scored higher than the paranormal group, and the paranoia group, *p* = .002, scored higher than the low all group on CC. The paranoid group had higher scores than the low all, *p* = < .001, and paranormal group, *p* = .004, on the NC subscale. Finally, the high all, *p* = .030, and paranoia group, *p* = .011, scored higher than the low all and paranormal group, high all: *p* = .009; paranoia group: *p* = .003, on CSC.

#### Effect of current mental health diagnosis between clusters on attributional styles

The main effect of cluster on all ASQ subscales was nonsignificant for people who did not report a mental health diagnosis.

For people who did report a diagnosis, the main effect of cluster on positive internalization (*M* = 4.761), *SE* = .083, *F* (3, 149) = 3.026, *p* = .031, η_p_^2^ = .057, and SSB (*M* = .025), *SE* = .130, *F* (3, 149) = 2.805 , *p* = .042, η_p_^2^ = .053, of the ASQ was significant, but not negative internalization. No cluster differences were seen across positive internalization and SSB for those with a reported mental health diagnosis after performing Bonferroni‐corrected post hoc comparisons.

## DISCUSSION

The aims of this article were twofold: to understand how unusual beliefs co‐occur in a general population sample and to discern whether distinctive profiles of beliefs would differ on cognitive styles. Magical ideation, paranormal beliefs, and paranoid thoughts were selected as the to‐be‐grouped unusual beliefs due to their commonality in general population samples (Bell & O'Driscoll, 2018; Tobacyk & Wilkinson, 1990). Metacognitive beliefs and attributional styles were chosen as outcome measures in this article as they represent cognitive styles commonly experienced by patients with delusions (So et al., 2015; Startup et al., 2016). We surmised that finding differences between unusual belief profiles on cognitive styles would provide evidence that biases co‐occur with unusual beliefs prior to receiving a clinical diagnosis of delusions.

We hypothesized that magical ideation, paranormal beliefs, and paranoid thoughts would form four meaningful participant groups. K‐means clustering produced four groups that best fit the data, in line with our hypothesis and previous schizotypy research (Barrantes‐Vidal et al., 2003; Suhr & Spitznagel, 2001a, 2001b): (a) low on all beliefs (low all), (b) high on all beliefs (high all), (c) high on paranormal beliefs in comparison to all other beliefs (paranormal group), and (d) high on paranoid beliefs compared to all other beliefs (paranoid group). Age and gender did not differ significantly between clusters. Mental health help‐seeking within the past 6 months was equally as likely to occur in the high all cluster as the paranormal cluster, and more common in these groups than the low all cluster; a pattern reflected in a recent meta‐analysis (Bhavsar et al., 2018). Expressing high levels of unusual beliefs or experiencing paranoid beliefs appears to make it likely that help will be sought for mental health difficulties. It suggests that these profiles of unusual beliefs are accompanied with a sufficient magnitude of distress that people seek help (Beattie et al., 2021; Byrne et al., 2015; Muñoz‐Negro et al., 2019; Thalbourne & Delin, 1994; Thalbourne & French, 1995; Varghese et al., 2011). On the other hand, the low all cluster appears to reflect a healthy community sample in this study. Supporting these ideas, participants in the paranormal group reported mental ill health to a lesser degree than those in the high all and paranoid clusters. Therefore, paranormal beliefs, at least in our sample, appear to be more psychologically adaptive than paranoid beliefs. Unusual belief experience is largely embedded in sociocultural contexts, influencing an individual's perception of the world, their own thoughts, and the intensity of their beliefs (Dutta et al., 2007). This is supported by previous research which has suggested that the increasingly cultural acceptability of paranormal beliefs provides a positive context for those who hold them (Castro et al., 2014; Cella et al., 2012; Drinkwater et al., 2017). For example, a 2013 Australian opinion poll showed 88% of surveyed people believed that paranormal phenomena exist, 70% of people claimed they had personal experience with anomalous phenomena, 50% believed in spirits and ghosts, and 40% believed in UFOs and aliens (Angel, 2014). This implies that at least for general population samples, paranormal beliefs may not be considered as “unusual” as originally claimed. However, this requires further investigation.

No distinctive profile was shown for magical beliefs. This was a curious finding considering that magical ideation has been demonstrated as a strong indicator of delusion proneness (Chan et al., 2015). Paranormal beliefs could be more related to belief conviction (Irwin, 2012) and paranoid beliefs associated with stronger affect (Freeman et al., 2011), which could partially explain the distinct profile of paranoia and paranormal beliefs, but not magical ideation. However, it could also be that magical ideation may be moderating the expression of other unusual beliefs. On one hand, magical ideation could potentially interact with distress to produce paranoia. Alternatively, magical beliefs may provide a framework for one to make sense of anomalous experiences, which may dull potential distress associated with unusual beliefs (Bell et al., 2007). It would be interesting for future studies to examine whether magical ideation may act as a moderating variable for the experience of other unusual beliefs and distress. As discussed in the introduction, magical ideation may also be related to paranoid and paranormal beliefs from a psychometric perspective, in overlapping items, as well as conceptually. Whether magical ideation provides the self‐referential content for other beliefs, or moderates their presentation, further research needs to consider whether magical ideation does have a distinct profile in the general population. While we included mental health help‐seeking and diagnosis as a proxy for distress, future studies need to include a self‐report measure of current psychological distress or affect to assist in understanding magical ideation's role in paranoia.

We hypothesised that people with stronger unusual beliefs would show maladaptive metacognitive styles. Our findings broadly appeared to be consistent with this hypothesis. In line with previous research, people who reported a mental health diagnosis had stronger positive beliefs about worry, negative beliefs about uncontrollability and danger of thoughts, and heightened awareness of their thinking (Cartwright‐Hatton & Wells, 1997; Papageorgiou & Wells, 2003). For the effect of cluster on metacognition, the high all and high paranoid clusters had more maladaptive metacognitions when compared with people in the low all and high paranormal clusters. The high all and high paranoid clusters did not differ on their metacognitive beliefs nor did the low all and paranormal clusters, suggesting that paranoid beliefs are associated with maladaptive processing around worry in a similar fashion to holding a broad spectrum of unusual beliefs. The findings for paranoia are consistent with prior clinical samples, providing further evidence that distress and mental ill health are more prevelant in people who experience greater paranoia.

Maladaptive metacognitive beliefs could bias people to perceive threat under ambiguous conditions (Wells & Matthews, 1996). Threat sensitivity is increased in people with emotional disorders, those who are exclusively paranoid, and those who have both magical and paranoid thinking (Freeman, 2007; Karcher & Shean, 2012). Less harmful metacognitive styles were shown in people who predominantly hold paranormal beliefs (paranormal group), which suggests that they are less psychologically harmful metacognitively than heightened paranoia or the endorsement of multiple unusual beliefs (Schofield & Claridge, 2007). Paranormal beliefs may not intrude in the perception of everyday life ambiguous experiences in the same manner as paranoid beliefs. Rather, paranormal beliefs could operate in a similar manner to magical beliefs in providing explanations for occurrences which are abstract, uncontrollable, and unseen (Subbotsky, 2010). Future research needs to consider the degree to which explanations of ambiguous circumstances are associated with different beliefs and the threshold for threat perception under such conditions.

We hypothesized that people with stronger unusual beliefs would show internal attribution biases for positive events, external attribution bias for negative events, and an exaggerated SSB in comparison to nonbelievers. Those in the high all and paranoid clusters were more likely to externalize the cause of positive events in comparison to people in the low all cluster, who were more likely to attribute the cause of positive events to themselves. A more pronounced SSB was shown for persons in the paranormal cluster compared to the Paranoid cluster. People who reported a mental health diagnosis were more likely to internalize negative events, externalize positive events, and show a diminished SSB, which is consistent with depressive attributional styles (Anderson et al., 1994; Peterson & Seligman, 1984). Although the effect of cluster on the ASQ was not dependent on mental health diagnosis, our exploratory analyses revealed that the effect of clusters on attributional styles was only prevalent for those people with a mental health diagnosis. It appears as though clinically significant psychopathology in conjunction with holding unusual beliefs may be driving the attributional style differences in our sample.

We expected that people with stronger unusual beliefs, particularly paranoia, would show an exaggerated SSB and externalization of negative events bias (Bentall et al., 2009; Chadwick et al., 2005; Gawęda, Prochwicz, et al., 2015; So et al., 2015). Our results did not support this: Reduced SSB and internalization of negative events were largely present in those people who hold multiple unusual beliefs concurrently, and stronger paranoid beliefs. Rather, persons with more paranormal beliefs held SSB and internalization of positive events similar to nonbelievers, reflecting attributional styles present in general population samples (Campbell & Sedikides, 1999; Mezulis et al., 2004). Attributing negative events to self and positive events to others in the high all and paranoid clusters appears to represent more depressive attributional styles (Humphreys & Barrowclough, 2006). This is supported by our findings for people who reported a mental health diagnosis in our sample, where 40% of these people reported either depression or comorbid anxiety and depressive disorders. paranoid‐depressive negative self‐attributions, known as “bad me,” describes a person believing they deserve and are personally responsible for persecution and malevolence they perceive, and have been reported in people who experience paranoia (Chadwick et al., 2005). Our research shows a depressive‐paranoia style of attribution in those people with ill mental health who hold strong unusual beliefs, and comparatively stronger paranoid beliefs. Importantly, maladaptive attributional styles are not unique to delusions as characterized by schizophrenia spectrum disorders (Peterson & Seligman, 1984). The hypothesized psychosis continuum model assumes that the experience of unusual beliefs in community samples that are associated with particular cognitive styles will lead to high risk of psychosis (Verdoux & Van Os, 2002). It could also be the case that attributional style could be associated with depression, where high levels of delusion symptoms, particularly paranoia, are also present (Moritz et al., 2017; Tennen et al., 1987). Future research could usefully include depression measures to assist in clarifying attributional styles.

There are several limitations in this article that require addressing. First, we did not include a measure of current mood state or distress; both the MCQ‐30 and ASQ are related to depression and anxiety (Peterson et al., 1982; Wells & Cartwright‐Hatton, 2004). This limitation is somewhat abated as current mental health diagnosis was addressed. Further, the ASQ is an older measure of attributional style that does not distinguish between types of externalization (i.e., due to a specific person or circumstance; Kinderman & Bentall, 1997). Although the outcomes of this study may have provided more nuanced results, the findings of the current article are in line with research using the ASQ, and the broader attributional styles literature. Negative affect (Sellers et al., 2018), threat sensitivity (Freeman, 2007), low self‐esteem (Bentall et al., 1994), and disruptions to interpersonal functioning (Hajdúk et al., 2019) have been related to paranoia pathology, and reflect important constructs for future studies to capture.

In conclusion, grouping participants based on multiple unusual beliefs has provided insight into how unusual belief profiles differ on cognitive styles. This article demonstrates that maladaptive thoughts about worry were related to higher endorsement of multiple unusual beliefs and paranoid thoughts. It also found that attributional biases were complicated by current mental health diagnosis in the sample. The results suggest that causal attributions associated with unusual beliefs may also be related to psychopathology outside of the schizophrenia spectrum disorders. Future research should investigate how unusual beliefs co‐occur to provide a more accurate representation of delusion‐proneness in general population samples.

## CONFLICT OF INTEREST

The authors disclose that there is no conflict of interest.

## ETHICS STATEMENT

This study was approved by the University of Wollongong Social Science and Humanities Human Research Ethics Committee (#2018/431), and informed consent was provided by all participants.

## Data Availability

The data that support the findings of this study are available from the corresponding author, EPC, upon reasonable request.
